# Two new combinations in *Euploca* Nutt. (Heliotropiaceae, Boraginales) and a conspectus of the species of the Guiana Shield area

**DOI:** 10.3897/phytokeys.61.6260

**Published:** 2016-02-25

**Authors:** Christian Feuillet

**Affiliations:** 1 Department of Botany, MRC–166, National Museum of Natural History, Smithsonian Institution, P.O. Box 37012, Washington, DC 20013-7012, USA; 2 Current address: Department of Botany and Plant Pathology, 2082 Cordley Hall, Oregon State University, Corvallis OR 97331-2902, USA

**Keywords:** Boraginales, Brazil, Euploca, Guiana Shield, Guianas, Heliotropiaceae, Venezuela

## Abstract

*Heliotropium foliatum* and *Tournefortia humilis* are transferred to *Euploca* Nutt. respectively as *Euploca foliata* **comb. n.** and *Euploca humilis* **comb. n.** A collection from Guyana has been recently identified as *Euploca humistrata*, a species previously considered a Brazilian endemic. A collection from French Guiana documents for the first time the species in that country. A key to the species of the Guiana Shield area is given. The species of *Euploca* from the Guiana Shield are listed with synonymy and a brief description: *Euploca filiformis*, *Euploca humilis*, *Euploca humistrata*, *Euploca lagoensis*, *Euploca polyphylla*, *Euploca procumbens*.

## Introduction

The genus *Euploca* Nuttall (1836: 189) was created for the new *Euploca convolvulacea* Nutt. (1836: 190). Between 1848 and 1951, four species and two infra specific names were published that are currently considered to be synonyms of *Euploca convolvulacea*. *Euploca convolvulacea* was transferred to *Heliotropium* L. (Linnaeus 1753, 1: 130) by Gray (1857: 403). Recently Hilger and Diane (2003) reevaluated the classification of the Heliotropiaceae Schrad. (Schrader 1819: 192) on the base of molecular data of nuclear ITS1 and plastidal *trn*LUAA intron sequences. One of their conclusions is that Heliotropium sect. Orthostachys (R.Br.) G.Don (Brown 1810: 493; Don 1838: 361), *Schleidenia* Endl. (Endlicher 1839: 646), and *Hilgeria* Förther (1998: 132) constitute a separate genus whose earlier available name is *Euploca*. Their analysis indicates that *Ixorhea* Fenzl (1886: 287) is sister to the rest of the family consisting of two clades: one composed of *Euploca* and genus *Myriopus* Small (1933: 1131) resurrected to accommodate the species of Tournefortia sect. Cyphocyema I.M.Johnst. (Johnston 1930: 72), the other one of *Heliotropium* and *Tournefortia* L. (1753, 1: 140). Morphologically *Euploca* and *Myriopus* differ from *Heliotropium* and *Tournefortia* by the shape and structure of the fruits and their curved embryo. *Euploca* species are herbs or subshrubs and have usually dry fruits, in contrast *Myriopus* species are shrubs or passive climbers and have 4-lobed fleshy fruits. Hilger and Diane (2003: p. 47–49) made 20 new combinations in *Euploca*. Melo and Semir (2006; 2009) described three new species and made 12 new combinations. More new combinations were made: one by Luebert (Luebert et al. 2011), four by Degen and Melo (2012), and two by Feuillet (2012). There are probably more than 45 species of *Heliotropium* and *Schleidenia* awaiting a transfer to *Euploca*. Nevertheless, the nomenclature of the species of *Euploca* in the Guiana Shield region should be stable after the new combination *Euploca humilis* given below.

## Methods

I studied the literature and type specimens. In the citation of types, “photo” refers to a print deposited in herbaria and “scan” refers to a picture posted online, available directly through herbarium sites, or sites like JSTOR or Europeana.

For the purpose of this paper, the Guiana Shield area is defined as the Venezuelan Guayana, the Guianas (Guyana, Surinam and French Guiana), and the part of Brazil north of the Amazon River and east of the Rio Negro.

## Nomenclature and new combinations

### Euploca

Taxon classificationPlantaeBoraginalesHeliotropiaceae

Nutt., 1836

Euploca Nutt., Trans. Amer. Phil. Soc., ser. 2, 5: 189. 1836.
Heliotropium [unranked] Orthostachys R.Br., Prodr.: 493. 1810. Type species. Heliotropium foliatum R.Br. (lectotype designated by Johnston 1928, pg. 46) [= Euploca foliata (R.Br.) Feuillet, PhytoKeys 61: 103. 2016.].
Preslaea Mart., Nov. Gen. Sp. 2: 75, t. 164. 1827; non PresliaOpiz 1824 (LAMIACEAE). Type species. Preslaea paradoxa Mart. [= Euploca paradoxa (Mart.) J.I.M.Melo & Semir].
Orthostachys (R.Br.) Spach, Hist. Nat. Vég. 9: 32. 1838. [“1840” see Stafleu and Cowan (1985: TL2 vol. 5: 767)] Type species. Based on Heliotropium [unranked] Orthostachys R.Br.
Heliotropium sect. Orthostachys (R.Br.) G.Don, Gen. Syst. 4: 361. 1838. Type species. Based on Heliotropium [unranked] Orthostachys R.Br.
Schleidenia Endl., Gen. Pl. 646. 1839. Type species. Based on Preslaea Mart.
Heliotropium subgen. Orthostachys (R.Br.) Rchb., Deut. Bot. Herb.-Buch: 112. 1841. Type species. Based on Heliotropium [unranked] Orthostachys R.Br.
Heliotropium sect. Euploca (Nutt.) A.Gray, Proc. Amer. Acad. Arts 10: 49. 1874. Type species. Based on Euploca Nutt.
Heliotropium sect. Orthostachys subsect. Axillaria I.M.Johnst., Contr. Gray Herb. 81: 47. 1928. Type species. Heliotropium paradoxum (Mart.) Gürke, in Engl. and Prantl, Nat. Pflanzenfam. 4(3a): 97. 1893; non Vatke 1875. (lectotype designated by Förther 1998, pg. 135) [= Euploca paradoxa (Mart.) J.I.M.Melo & Semir].
Heliotropium sect. Orthostachys subsect. Bracteata I.M.Johnst., Contr. Gray Herb. 81: 47. 1928. Type species. Heliotropium bracteatum R.Br., Prodr.: 493. 1810.
Heliotropium sect. Orthostachys subsect. Ebracteata I.M.Johnst., Contr. Gray Herb. 81: 47. 1928. Type species. Heliotropium procumbens Mill., Gard. dict.. ed. 8: 10. 1768; non Kunth 1818. [= Euploca procumbens (Mill.) Diane & Hilger].
Hilgeria Förther, Sendtnera 5: 132. 1998. Type species. Hilgeria hypogaea (Urb. & Ekman 1929: 105) Förther [= Euploca hypogaea (Urb. & Ekman) Diane & Hilger].

#### Type species.

*Euploca convolvulacea* Nutt.

#### Description.

Herbs or subshrubs. Leaves alternate, simple, entire. Trichomes on a pedestal of distinctly enlarged foliar epidermis cells. Inflorescences of single, scorpioid cymes, or flowers solitary. Flowers perfect; calyx 5–toothed or 5–lobed, persistent; corolla white, small, 5–merous, lobe’s margin involute; stamens 5, included; nectar disc at the base of the ovary; ovary functionally 4–locular, locules 1-seeded, style terminal, undivided, or absent, the stigma cryptically or clearly apically 2- or 4-fid. Fruit a schizocarp, lobed or unlobed, dry, 2–4 bony 1-seeded nutlets, embryos curved.

#### Distribution.

North America to Argentina, Africa, Arabian Peninsula to Indochina, Australia.

### Euploca foliata

Taxon classificationPlantaeBoraginalesHeliotropiaceae

(R.Br.) Feuillet comb. nov.

urn:lsid:ipni.org:names:77153393-1

Heliotropium foliatum R.Br., Prodr.: 493. 1810.
Euploca foliata Type. Australia. Northern Territory: Carpentaria mainland opposite Grote Eyland, 4 Jan 1803, *R. Brown 2932* (lectotype, designated by Craven 1996, pg. 630: BM n.v. [scan!]; isotypes: K!, MEL n.v., NSW n.v.).

#### Type.

Based on *Heliotropium foliatum* R.Br.

#### Notes.

When they resurrected *Euploca*, Hilger and Diane (2003: 42) said that “summarizing all urges a formal taxonomic recombination of EUPLOCA, including all species of Heliotropium section Orthostachys, *Schleidenia*, and *Hilgeria* into the genus *Euploca*, the oldest available generic name in this group.” *Heliotropium foliatum* is the type of Heliotropium sect. Orthostachys (Johnston 1928: 46). Although it is recognized as a good species (example: Förther 1998: 195), *Heliotropium foliatum* was not transferred to *Euploca* until now. Robert Brown did not cite a type collection, only mentioning “v. v.” (=seen alive).

### Euploca humilis

Taxon classificationPlantaeBoraginalesHeliotropiaceae

(L.) Feuillet comb. nov.

urn:lsid:ipni.org:names:77153394-1

Tournefortia humilis L., Sp. Pl.: 141. 1753. Type. “Tournefortia foliis lanceolatis” pl. by Plumier, in Burman, Pl. Amer., 224, t. 227, f. 2. 1760 (lectotype designated by Miller in Cafferty and Jarvis 2004, pg. 804). Epitype: FRANCE. Martinique: Case Pilote, Feb 1868, *L. Hahn 416* (designated by Miller in Cafferty and Jarvis 2004, pg. 804: BM n.v.).

#### Type.

Based on *Tournefortia humilis* L.

#### Notes.

When Melo and Semir (2009: 289) transferred *Heliotropium ternatum* Vahl (1794, 3: 21) to *Euploca*, they did not cite synonyms and they overlooked the case of *Tournefortia humilis* L. There had been a doubt about the exact identity of the Linnean species (Johnston 1949: 136; Förther 1998: 190), but the typification of *Tournefortia humilis* L. by Miller (in Cafferty and Jarvis 2004: 804) placed clearly that species in the synonymy of *Heliotropium ternatum* Vahl (non *Heliotropium humile* Lam.). The epithet of *Tournefortia humilis* could not be transferred to *Heliotropium*, but there is no previous *Euploca humilis* and the priority rule dictates the adoption of the new combination (McNeill et al. 2012: Art. 11.4).

Because the name *Tournefortia humilis* L. or/and *Heliotropium humile* (L.) R.Br. ex Roem. & Schult. (1819, 4: 37; non Lam., 1791: 393) have been used in synonymy (Johnston 1928: 69; 1949: 135; and especially the recent revision of *Heliotropium* Förther 1998: 190, 234; and the typification Miller, in Cafferty and Jarvis 2004: 804); because it is a Linnean name; and because the authors of the new combination *Euploca ternata* (Melo and Semir 2009: 289) should have been aware of that name, there are little chances to make a successful proposal to conserve *Euploca ternata*.

### Conspectus of *Euploca* for the Guiana Shield region

The last treatment of *Heliotropium* (including *Euploca*) for the Guianas (Johnston 1935) included four of the six species treated below: *Heliotropium filiforme*=*Euploca filiformis*, *Heliotropium lagoense*=*Euploca lagoensis*, *Heliotropium procumbens*=*Euploca procumbens*, *Heliotropium ternatum*=*Euploca humilis*. Since then *Euploca humistrata* has been identified from a seasonally flooded savanna in Guyana, the distribution area of *Euploca lagoensis* includes French Guiana. Besides, *Euploca polyphylla* not present in the Guianas occurs in the Venezuelan Guayana (State of Bolívar). The species present in the Guiana Shield region are usually found on sand near water or in seasonally dry localities.

### Key to the species of *Euploca* in the Guiana Shield

**Table:** 

1	Flowers solitary; pedicels 1–3 mm long; corolla white and yellow	**2**
–	Inflorescences 1–18 cm long; flowers sessile or sub-sessile, or pedicels obvious; corolla white or yellow	**3**
2	Stem glabrous or puberulent; leaf blades glabrous or puberulent; corolla white, yellow at throat, lobes elliptic	***Euploca lagoensis***
–	Stem villose; leaf blades with long, stiff trichomes; corolla tube yellow, lobes white, ovate	***Euploca humistrata***
3	Petiole 4–15 mm long; raceme mostly geminate or ternate, ebracteate; stigma sessile, base as wide as the apex of the ovary, forming together a blunt cone	***Euploca procumbens***
–	Petiole 0.3–2 mm long; raceme single or paired, bracteate; stigma morphologically clearly separate from the ovary, looking together like a mushroom or like a head with a hat or with a clear style	**4**
4	Leaf blade Inflorescences always single; bract filiform to narrowly elliptic; calyx length greater than or less than half the corolla tube; corolla white; style 0.3–0.5 mm long	**5**
–	Inflorescences usually single and paired on the same plant; bracts ovate; calyx nearly as long as the corolla tube; corolla yellow; style 0.5–1 mm long	***Euploca polyphylla***
5	Stem relatively coarse and stiff; leaves drying rather light colored; inflorescences up to 20 cm long, with 1–2 cm long peduncle; calyx shorter than half the corolla tube; corolla 4–6 mm long, salverform; anthers apically joined; stigma on a short style	***Euploca humilis***
–	Stem wiry, very slender; leaves usually drying dark colored; inflorescences epedunculate, up to 10 cm long; calyx longer than the corolla tube; corolla 2–2.5 mm long, tube urceolate; anthers not joined at apex; stigma sessile	***Euploca filiformis***

### Euploca filiformis

Taxon classificationPlantaeBoraginalesHeliotropiaceae

(Lehm.) J.I.M.Melo & Semir, 2009

Euploca filiformis (Lehm.) J.I.M.Melo & Semir, Kew Bull. 64: 289. 2009.
Heliotropium filiforme Lehm., Gött. Gel. Anz. 3(152): 1515. 1817.
Euploca filiformis Type. Venezuela. Near Orinoco, *Bonpland and Humboldt 1202* [an error, most likely for *1203*] (lectotype, designated by Förther 1998, pg. 195: B-W 3246/1 n.v. [scan!]; isotypes MEL 233288 n.v., P! [2 sheets; scans!], P-Bonpl! [scan!]). [same type collection as next, but different holotype]
Heliotropium filiforme Kunth, Nov. Gen. Sp. (quarto ed.) 3: 86, t. 204. 1818, nom. illeg. non Heliotropium filiforme Lehm. 1817.
Euploca filiformis Type. Venezuela. Near Orinoco, *Bonpland and Humboldt 1203* (holotype P-Bonpl! [scan!]; isotypes B-Willd 3246/1 n.v. [scan!], MEL 233288 n.v., P! [2 sheets; scans!]. [same type collection as previous, but different holotype]
Heliotropium tenue Roem. & Schult., Syst. Veg. 4: 737. 1819.
Euploca filiformis Type. Brazil. Pará, *Sieber for Hoffmannsegg s.n.* (holotype B-WILLD-3248!; isotypes HAL n.v., MEL 233189 n.v., P!).
Preslaea stenostachya A.St.-Hil., Voy. Distr. Diam. 2: 434. 1833.
Euploca filiformis Type. Brazil. “Ad ripas fluminis Parahyba”, *St.-Hilaire B228* (lectotype, designated by Förther 1998, pg. 235: P! [scan!]; isotypes K!, M n.v., MPU!, P! [scan!]).
Heliotropium littorale Mart. ex Colla, Herb. Pedem. 4: 226. 1835.
Euploca filiformis Type. Brazil. “In arenosis maritimis”, *Martius s.n.* (holotype TO n.v.; isotype BM n.v.).
Euploca filiformis Note. placed in the synonymy fide Förther 1998 who saw the isotype at BM.
Heliotropium pusillum Colla, Herb. Pedem. 4: 227. 1835.
Euploca filiformis Type. Brazil. *Martius s.n.* (holotype TO n.v.).
Heliotropium helophilum Mart., Flora 21(2), Beibl. 4: 85. 1838.
Euploca filiformis Type. Brazil. “Propre Cujaba”, 1837, *Herb. Fl. Bras. 165, n°267* (lectotype, designated by Förther 1998, pg. 199: BR n.v. [scan!]; isotypes E n.v. [scan!], FI-W!, G-DC!, GH n.v., L! [2 sheets], LE n.v. [2 sheets], M n.v., P! [2 sheets; scans!], W n.v.).
Schleidenia stenostachya (A.St.-Hil.) DC., Prodr. 9: 558. 1845. Type. Based on Preslaea stenostachya A.St.-Hil.
Schleidenia filiformis (Lehm.) Fresen., Fl. Bras. 8(1): 40. 1857. Type. Based on Heliotropium filiforme Lehm.
Heliotropium stenostachyum (A.St.-Hil.) Gürke, in Engler and Prantl, Nat. Pflanzenfam. 4(3a): 97. 1893. Type. Based on Preslaea stenostachya A.St.-Hil.

#### Type.

Based on *Heliotropium filiforme* Lehm.

#### Description.

Annual herb or subshrub, up to 0.25 m tall. Stems slender, erect or decumbent, up to 0.4 m long, sparingly strigose. Leaves: petiole 0.1–0.2 cm long, slender; lamina linear or elliptic or lanceolate or oblanceolate, 1–2.5 × 0.15–0.35 cm, apex acute, base acute, margin entire, both surfaces sericeous to strigose. Inflorescences epedunculate, slender scorpioid cymes, up to 15 cm long, bearing scattered bracts, filiform or subulate, 0.1–0.2 cm long, glabrescent. Flower sub–sessile: calyx longer than the corolla tube, lobes, 1.5–2 mm long at anthesis, glabrescent; corolla white, tubular, outside strigose, inside glabrescent, tube narrowed in the middle, lobes ovate with broadly open sinuses, about 1 mm long; stamens sessile, anther with an apical appendage, weakly coherent; ovary subglobose or globose, strigose; style lacking or nearly so; stigma sessile or sub–sessile, fertile base much wider than the apex of the ovary. Fruit on 0.5–1 mm long pedicel, depressed–globose; nutlets almost 1.5 mm long.

#### Distribution.

Venezuela (Amazonas, Bolívar), Guyana, Surinam, French Guiana, Brazil (Amapá, Amazonas, Pará, Roraima); also in Belize, Mexico, southeastern USA and from Trinidad to eastern Bolivia and Paraguay.

#### Selected specimens studied.

**Guyana**: Cuyuni River, Matope Falls, 4 June 1952, *Forest Department 6940 (Fanshawe 3376)* (K, NY); Upper Takutu-Upper Essequibo, Dadanawa, 24 Oct 1957, *Cook 22* (K, NY). **Surinam**: near Coppename River, Central Suriname Nature Reserve, 4°25'N, 56°31'W, 50–75 m, 22 Feb 2004, *Clarke 11037* (US); near Ulemari River, 13 km upstream from confluence with Litani River, 3°13'N, 54°15'W, 150 m, 2 Apr 1998, *Hammel 21275* (MO, US). **French Guiana**: Itany River, Saut upstream from Touinké, 26 Nov 1977, *Cremers 5119* (CAY); Oyapock River, Sauts Fourmi, 18 Nov 1984, *Granville 6916* (CAY, P).

### Euploca humilis

Taxon classificationPlantaeBoraginalesHeliotropiaceae

(L.) Feuillet comb. nov.

urn:lsid:ipni.org:names:77153394-1

Tournefortia humilis L., Sp. Pl. 1: 141. 1753. Type. “Tournefortia foliis lanceolatis” in Plumier in Burman, Pl. Amer., 224, t. 227, f. 2, 1760 [http://biodiversitylibrary.org/page/779658] (lectotype designated by Miller in Cafferty and Jarvis 2004: 804). Epitype: France. Martinique: Case Pilote, Feb 1868, *L. Hahn 416* (designated, by Miller in Cafferty and Jarvis 2004, 804: BM n.v.)).
Heliotropium ternatum Vahl, Symb. Bot. 3:21. 1794.
Euploca humilis Type. [Jamaica], “*in India occidentali*” (holotype C n.v. [scan!; microfiche 37: I, 3; photo GH!]).
Heliotropium hirtum Lehm., Neue Schriften Naturf. Ges. Halle 3(2): 10. 1817. Type. Venezuela. Cumana, *Humboldt and Bonpland s.n.* (lectotype, designated by Förther 1998, pg. 199: B-WILLD 3247!; isotypes MEL 233292 n.v., P! [scan!], P-Bonpl! [scan!]) [same type collection as Heliotropium hispidum Kunth (see below), but a different holotype].
Heliotropium demissum Roem. & Schult., Syst. Veg. ed 15bis 4: 37. 1819. Type. nom. illeg. renaming of Tournefortia humilis L.
Heliotropium humile (L.) R.Br. ex Roem. & Schult., Syst. Veg. ed 15bis 4: 37. 1819; non Lam. 1791.
Euploca humilis Type. nom. illeg. in syn. of previous.
Heliotropium hispidum Kunth, Nov. Gen. Sp. (quarto ed.) 3: 87. 1818; illeg. not Lehm. 1817. Type. Venezuela. near Cumana, Sep, *Humboldt and Bonpland s.n.* (holotype P-Bonpl! [scan!]; isotypes B-WILLD 3247!, MEL 233292 n.v., P! [scan!]) [same type collection as Heliotropium hirtum Lehm. (see above), but a different holotype].
Pioctonon ternatum (Vahl) Raf., Sylva Tellur.: 88. 1838. Type. Based on Heliotropium ternatum Vahl
Pioctonon antillanum Raf., Sylva Tellur. 88. 1838. Type. nom. illeg. renaming of Tournefortia humilis L.
Tournefortia incana (G.Mey.) G.Don, Gen. Hist. 4: 368. 1838; non Lam. 1791. Type. Based on Messerschmidia incana G.Mey.; nom. inval.
Tournefortia meyeri DC., Prodr. 9: 530. 1845. Type. Renaming of Messerschmidia incana G. Mey.; nom. inval.
Heliotropium fruticosum var. ternatum (Vahl) DC., Prodr. 9: 542. 1845. Type. Based on Heliotropium ternatum Vahl
Heliotropium oaxacanum DC., Prodr. 9: 543. 1845.
Euploca humilis Type. Mexico. Oaxaca, Aug 1834, *Andrieux 205* (holotype G-DC !; isotypes FI-W n.v. [fragment GH n.v.], P!, W n.v.).
Heliophytum passerinoides Klotzsch, in Rich. Schomburgk, Reis. Br.-Guiana 3: 1152. 1849 [“1848”]; nom. nud.
Euploca humilis Type. Based on Guyana. Rupununi: May 1842, *Schomburgk 573* (B†).
Schleidenia hispida (Kunth) Fresen., Fl. Bras. 8(1): 37. 1857. Type. Based on Heliotropium hispidum Kunth
Schleidenia fumana Fresen., in Mart.: Fl. Bras. 8, 1: 40. 1857.
Euploca humilis Syntype. Brazil. Minas Gerais, *Vauthier s.n.* (G n.v.). Type : Brazil. “ad Formigas et Ribeirao Catinga”, *Pohl 565 (= 3081 bzw. distr. nr. 1588)* (lectotype, designated by Förther 1998, pg. 235: W n.v., photo MSB-55474; isotypes B† [photo F-17322, GH!, NY!, US!], F-874797 n.v. [scan!], GH, K!, M n.v., [as s.n.] W n.v.).
Heliotropium fumana (Fresen.) Gürke, in Engler and Prantl: Nat. Pflanzenf. 4, Abt. 3a: 97. 1893. Type. Based on Schleidenia fumana Fresen.
Heliotropium mexicanum Greenm., Proc. Amer. Acad. Arts 33(25): 484. 1898; illegitimate: not Sessé & Moç. 1888.
Euploca humilis Syntypes: Mexico. *Dugès s.n.*; *Pelmer 31, 98*; *L.C. Smith 209, 391*; *Alvarez 750*; *Pringle 672*6. Type. Mexico. Iron Mtn, near Durando, June 1897, *Palmer 141* (lectotype, designated by Frohlich 1981, pg. 100: GH n.v.; isotypes BM n.v., F n.v., M n.v., MICH n.v. [scan!], MO!, NY!, S n.v., US!).
Heliotropium strictissimum sensu N.E. Br. 1901; not A. DC. 1845.
Heliotropium ternatum var. fumana (Fresen.) I.M.Johnst., J. Arnold Arb. 16:62. 1935. Type. Based on Schleidenia fumana Fresen.
Heliotropium greenmanii Wiggins, Contr. Dudley Herb. 4:22. 1950. Type. Based on Heliotropium mexicanum Greenm.
Euploca ternata (Vahl) J.I.M.Melo & Semir, Kew Bull. 64: 289. 2009. Type. Based on Heliotropium ternatum Vahl

#### Type.

Based on *Tournefortia humilis* L.

#### Description.

Suffrutescent herbs up to 30 cm tall; stems stiff, erect or decumbent, 10–50 cm long, with abundant, loosely appressed trichomes. Leaves: petiole subnul–2 mm long, thin; lamina lanceolate to linear, revolute, 0.5–3 × 0.1–0.8 cm, apex acute, base acute, margin +/- revolute, adaxially sericeous, abaxially tomentose, trichomes base very large and looking calcified. Inflorescences terminal or axillary, up to 20 cm long, stiff scorpioid cymes, single, peduncle 1–2 cm long, with scattered linear or narrowly elliptic bracts, 2–3 mm long, sericeous. Flower subsessile: pedicel 0.5–1 mm long, calyx 2–3 mm long, deeply lobed, lobes unequal, shorter than 1/2 the length of the corolla tube, becoming twice as large at maturity, sericeous in and out; corolla white, strigose outside, tube narrow, 2–2.7 mm long, throat yellow, lobes spreading, 1.3–2 mm long, ovate, sinuses rounded and plicate; stamens subsessile, anthers ovate with short, obtuse, hairy, apical long appendages which are apically joined; ovary globose, 0.3–0.4 mm diam., glabrous; style short but obvious, about 0.5 mm long; stigma about as large as the ovary, split at apex. Fruit depressed globose, 4–lobed, 1.5–1.8 mm diam., strigose or hirsute; nutlets 1–1.5 mm long.

#### Distribution.

Venezuela (Bolívar) and Guyana; also Central America, Mexico, and the West Indies Southward to southern Brazil.

#### Selected specimens studied.

**Guyana**: Potaro-Siparuni, Iwokrama, Kurupukari Falls, 4°40'N, 58°40'W, 100 m, 9 Dec 1994, *Mutchnick 595* (BRG, CAY, L, MO, NY, US); Upper Takutu-Upper Essequibo, near Sand Creek, Baboon Hill, 3°00'N, 59°31'W, 120–150 m, 21 June 1989, *Gillespie 1801* (BRG, CAY, L, MO, US); near Sand Creek, Sep 1948, *Wilson-Browne 128* (NY).

### Euploca humistrata

Taxon classificationPlantaeBoraginalesHeliotropiaceae

(Cham.) J.I.M.Melo & Semir, 2009

Fig. 1

Euploca humistrata (Cham.) J.I.M.Melo & Semir, Kew Bull. 64(2): 288. 2009
Heliotropium humistratum Cham., Linnaea 4: 462. 1829.
Euploca humistrata Type [fide Förther 1998: 200]: Brazil. Minas Gerais: Faz de Piedade, 1818, *Sellow s.n.* (holotype LE n.v.; isotypes B† [fragment GH n.v.; photo F-17325, GH, NY, US], G-DC!, HAL n.v. [scan!], K!).
Schleidenia humistrata (Cham.) Fresen., Fl. Bras. 8(1): 34. 1857. Type: Based on Heliotropium humistratum Cham.

#### Type.

Based on *Heliotropium humistratum* Cham.

#### Distribution.

Prostrate herbs. Stems villose, trichomes hyaline, somewhat ferruginous around the nodes. Leaves: petiole short; lamina lanceolate, 0.5–0.8 × 0.15–0.2 cm, apex acute, base attenuate, margin entire, both surfaces villose. Inflorescence: solitary, supra-axillary flowers, on 1–1.5 mm long pedicel. Flowers: calyx as long as the corolla tube, lobes, 2.2–2.7 mm long at anthesis, puberulent on both sides; corolla tube yellow with white lobes, outside strigose, inside pubescent, tube more or less cylindric, lobes ovate with narrow sinuses, about 1 mm long; stamens sessile, anther with a small appendage; ovary ca. 0.3 mm diam., globose, glabrous; style lacking; stigma sessile, widely conical. Fruit on 1.2–2 mm long pedicel, subglobose, 1.5 mm diam., glabrous, rostrate; nutlets about 1.2 mm long.

#### Distribution.

Guyana; previously only known from a few localities outside the Guiana Shield, Brazil (Ceará, Goiás, Minas Gerais), Venezuela (Apure). In our area it is known only by GUYANA, Upper Takutu–Upper Essequibo, W of the Kanuku Mtns, savanna near Mountain Point, 27 Feb–5 Mar 1985, fl, *C. Feuillet 1632* (CAY).

#### Notes.

Melo and Semir (2009: 288) cited the type as “*Oliveira 239* (holotype BHCB!; isotype BHMH!)”. This is obviously a misunderstanding. Melo and Semir (2010: 117) cited the holotype, *Sellow s.n.*, as being at GH. In fact at GH is a fragment of the isotype, now destroyed, that was at B. Förther (1998: 200) cites the holotype at LE.

**Figure 1.**
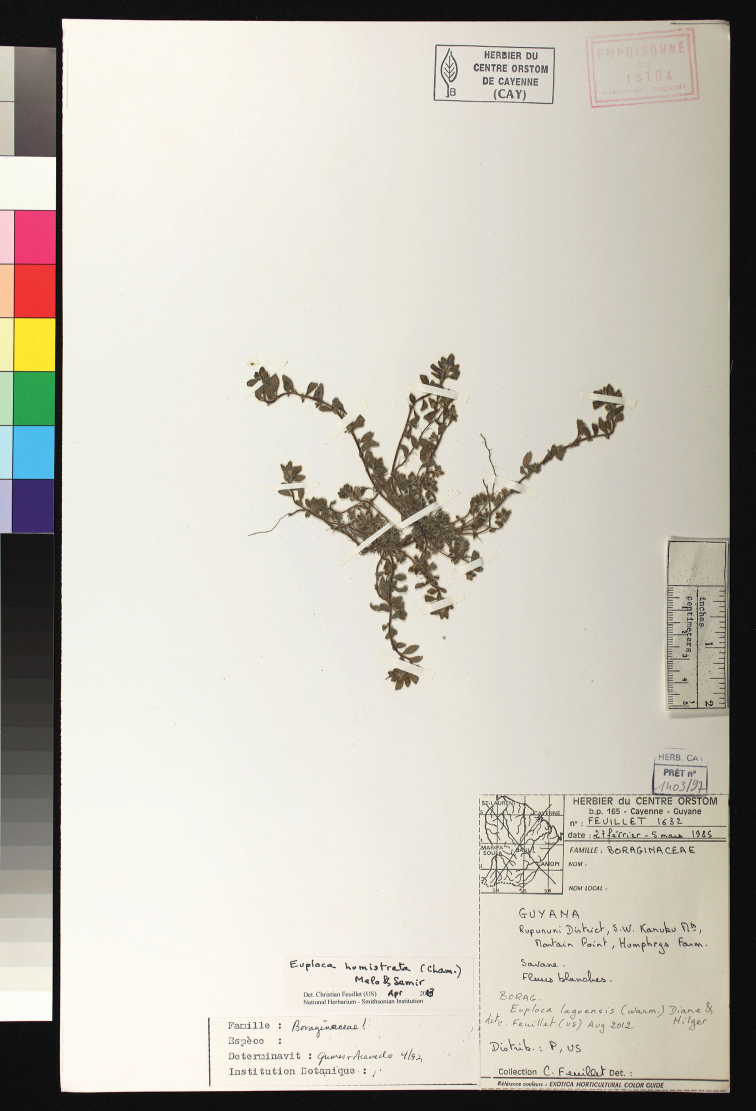
*Euploca humistrata*. Guyana, Upper Takutu-Upper Essequibo, Rupununi savanna, savanna near Mountain Point, 27 Feb–5 Mar 1985, *Feuillet 1632* (CAY). Picture by Ingrid Lin at US.

### Euploca lagoensis

Taxon classificationPlantaeBoraginalesHeliotropiaceae

(Warm.) Diane & Hilger, in Hilger and Diane, 2003

Fig. 2

Euploca lagoensis (Warm.) Diane & Hilger, in Hilger and Diane: Bot. Jahrb. Syst. 125 (1): 48. 2003.
Schleidenia lagoensis Warm., Kjoeb. Vidensk. Meddel 1867: 15. 1868.
Euploca lagoensis Type: Brazil. Minas Gerais: Lagoa Santa ad ripam lacus, 5 Nov 1864, *Warming s.n.* (holotype C n.v. [scan!; photos F-21971, M, MO]; isotypes GH n.v. [fragment], P! [scan!]).
Schleidenia subracemosa Warm., Kjoeb. Vidensk. Meddel 1867: 15. 1868.
Euploca lagoensis Type: Brazil. Minas Gerais: Lagoa Santa, 28 Jan 1866, *Warming s.n.* (holotype C [photo F-21972, GH, US]; isotype P! [scan!]).
Heliotropium lagoense (Warm.) Gürke, in Engler and Prantl, Nat. Pflanzemf. 4(3a): 97. 1893. Type: Based on Schleidenia lagoensis Warm.
Heliotropium trinitense Urb., Symb. Ant. 7(3): 350. 1912.
Euploca lagoensis Type: Trinidad. in Piarco Savannah, 10 May 1895, *Lunt 6030* (holotype B †; lectotype, designated by Förther 1998, pg. 225: S n.v. [scan!]; isotypes [fragments of holotype GH n.v. and lectotype GH n.v.]).

#### Type.

Based on *Schleidenia lagoensis* Warm.

#### Description.

Annual herbs, up to 15 cm tall, stems decumbent or prostrate, up to 30 cm long, glabrous or with scattered slender appressed trichomes. Leaves: petiole about 1 mm long, glabrous; lamina oblanceolate, 0.5–1.5 × 0.1–0.3 cm, apex acute to acuminate, base attenuate, margin entire, ciliate, adaxially glabrous, abaxially glabrous to puberulent. Flowers single, extra–axillary on leafy stems: pedicel up to 3 mm long, calyx of 5 lanceolate or cuneate, unequal lobes, 1.5–2.5 mm long at anthesis, puberulent in and out; corolla white, yellow at throat, tube swollen at base, 3–4 mm long, puberulent in and out, lobes ovate, 1–1.5 mm long, with sinus rounded, occasionally plaited with a minute lobule; stamens sessile, inserted about 0.5 mm from base, anthers joined, oblong, with an hairy apical appendage nearly as large as the anther; ovary obpyriform, about 0.5 mm diam.; style lacking or confused with the apex of the ovary; stigma sessile or subsessile, much larger at base; fruit rostrate, glabrous or nearly so, about 2 mm diam., obpyriform; nutlets ca. 1.5–2 mm long.

#### Distribution.

Venezuela (Bolívar), Surinam, French Guiana, and Brazil (Amazonas); also Greater Antilles and from Mexico to Brazil and eastern Bolivia. *Euploca lagoensis* is new to French Guiana. I have seen only few specimens from the Guiana Shield.

#### Selected specimens studied.

**Venezuela**, Bolívar, near San Carlos, Laguna de la Culebra, 6 Apr 1925, fl, *Pittier 11703* (P). **Surinam**, In 1885, *W.F.R. Suringar s.n.* (L); *Mennega 907* (L). **French Guiana**, Savane Maillard, 6 km SE de Tonate-Macouria, 4°58'N, 52°26'W, 25 Nov 1999, *Raynal-Roques and Jérémie 24694* (CAY, K, P, NY).

#### Note.

Melo and Semir (2010) cited the holotype as being at M. I do not think there is a specimen at M, only a photograph. Furthermore, if Warming worked in Munich, it was about 7 years after he described *Schleidenia lagoensis*. In the 1867 he was working in Copenhagen, I do not see any reason to reject Förther’s (1998) choice of the specimen at C as the holotype.

**Figure 2.**
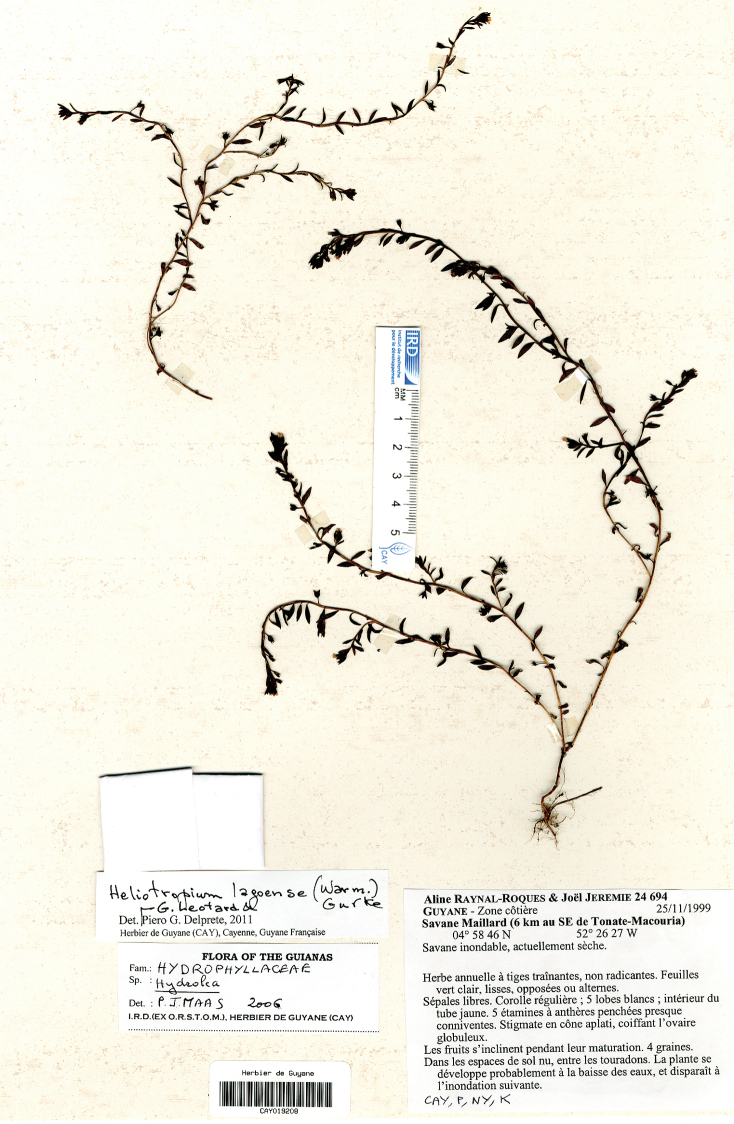
*Euploca lagoensis*. French Guiana, SE of Tonate-Macouria, Savane Maillard, 25 Nov 1999, *Raynal-Roques and Jérémie 24694* (CAY). (Picture by Piero Delprete at CAY).

### Euploca polyphylla

Taxon classificationPlantaeBoraginalesHeliotropiaceae

(Lehm.) J.I.M.Melo & Semir, 2009

Euploca polyphylla (Lehm.) J.I.M.Melo & Semir, Kew Bull. 64(2): 289. 2009.
Heliotropium polyphyllum Lehm., Neue Schriften Naturf. Ges. Halle 3(2): 9. 1817.
Euploca polyphylla Type (fide Förther 1998: 214). Venezuela. near Orinoco, *Humboldt s.n.* (holotype MEL 233310 n.v.; isotypes B-Willd 3252 n.v., P! [scan!], S 11-21939 n.v. [scan!]).
Heliotropium foliosum Roem. & Schult., Syst. Veg. 4: 737. 1819.
Euploca polyphylla Type. Venezuela. Orinoco, Apr 1800, *Humboldt and Bonpland 808* (holotype B-WILLD 3252-01 n.v.).
Preslaea linifolia A.St.-Hil., Voy. Distr. Diam. 2: 433. 1833.
Euploca polyphylla Type. Brazil. Cabo Frio, *St.-Hilaire 433* (lectotype, designated by Förther 1998, pg. 234: P!; isotypes B †, M n.v., MPU!, P! [2 sheets]).
Heliotropium bahiense DC., Prodr. 9: 544. 1845.
Euploca polyphylla Syntype: Brazil. *Blanchet 92* (G n.v., NY!, P!). Type. Brazil. Bahia, 1836, *Salzmann 368* (lectotype, designated by Förther 1998, pg. 181: G-DC n.v.; isotypes B †, F n.v., H n.v., HAL n.v., K!, M n.v., MPU!, P! [4 sheets]).
Heliotropium polyphyllum var. blanchetii DC., Prodr. 9: 544. 1845.
Euploca polyphylla Type. Brazil. Bahia, Serra Jacobina, 1837, *Blanchet 2651* (lectotype, designated by Förther 1998, pg. 214: G-DC n.v.; isotypes B† [photo F-17340, GH, NY, US], BM n.v. [fragment GH n.v.], BR n.v., F-520830 n.v. [photo F-57735], FI-W!, HAL n.v., K! [scan !], LE n.v., M n.v., NY!, P! [2 sheets], TUB n.v., US-292391!, W-118162 n.v.).
Schleidenia linifolia (A.St.-Hil.) DC., Prodr. 9: 558. 1845. Type. Based on Preslaea linifolia A.St.-Hil.
Schleidenia pubescens Fresen., Fl. Bras. 8(1): 35. 1857.
Euploca polyphylla Type. Brazil. Rio de Janeiro, *Gaudichaud [524*] (lectotype, designated by Förther 1998, pg. 236: P!; isotype B†).
Schleidenia polyphylla (Lehm.) Fresen., Fl. Bras. 8(1): 36. 1857. Type. Based on Heliotropium polyphyllum Lehm.
Schleidenia polyphylla var. blanchetii (DC.) Fresen., Fl. Bras. 8(1): 36. 1857. Type. Based on Heliotropium polyphyllum var. blanchetii DC.
Schleidenia bahiensis (DC.) Fresen., Fl. Bras. 8(1): 43. 1857. Type. Based on Heliotropium bahiense DC.
Heliotropium polyphyllum var. laevenworthii A.Gray, Syn. Fl. N. Amer. 2(1): 185. 1878.
Euploca polyphylla Syntypes: USA. Florida: Everglades, *Leavenworth s.n.*; *Palmer s.n.* (F). Type. USA. Florida: Everglades, 1877, *Garber s.n.* (lectotype, designated by Förther 1998, pg. 214: GH n.v.; isotype K n.v. [scan!]).
Heliotropium pubescens (Fresen.) Gürke, in Engler and Prantl, Nat. Pflanzenfam. 4(3a): 97. 1893. Type. Based on Schleidenia pubescens Fresen.
Heliotropium linifolium (A.St.-Hil.) Gürke, in Engler and Prantl, Nat. Pflanzenfam. 4(3a): 97. 1893; illegitimate: not Lehm. 1818. Type. Based on Preslaea linifolia A.St.-Hil.
Heliotropium laevenworthii (A.Gray) Small, Fl. S. E. U.S.: 1006. 1903. Type. Based on Heliotropium polyphyllum var. laevenworthii A.Gray
Heliotropium horizontale Small, Bull. New York Bot. Gard. 3: 435. 1905.
Euploca polyphylla Type. USA. Florida: 9–12 Nov 1903, *Small and Carter 742* (holotype NY! [photo GH]; isotypes F n.v., GH n.v. [fragment], US!).
Heliotropium polyphyllum var. horizontale (Small) R.W.Long, Rhodora 72: 33. 1970. Type. Based on Heliotropium horizontale Small

#### Type.

Based on *Heliotropium polyphyllum* Lehm.

#### Description.

Herb or subshrub. Stems prostrate or decumbent, sericeous. Leaves alternate or subopposite: petiole 0.03–0.1 cm long, sericeous; lamina narrow lanceolate or oblanceolate, 0.6–1.3 × 0.1–0.2 cm, apex acute, base cuneate, margin entire, both surfaces sericeous. Inflorescences single or paired, cymose, 1–15 cm long, bracts ovate, 0.3–0.4 cm long, sericeous inside, glabrous outside. Flower sub–sessile: calyx slightly shorter than the corolla tube, lobes, 3–4 mm long at anthesis, glabrous inside, sericeous outside; corolla yellow or white, tubular, outside sericeous, inside sericeous at throat, tube widened in the middle, lobes ovate-deltoid, with narrow sinuses, 2.5–3 mm long; stamens subsessile, anther with a long appendage; ovary subglobose, 4-sulcate, glabrous; style 0.3–0.6 mm long; stigma conical. Fruit subglobose, entirely covered by the calyx; nutlets almost 1.5 mm diam.

#### Distribution.

Venezuela (Bolívar), Brazil (Amapá, Amazonas, Pará); also USA (Florida), Bahamas, Venezuela, eastern Brazil.

### Euploca procumbens

Taxon classificationPlantaeBoraginalesHeliotropiaceae

(Mill.) Diane & Hilger, in Hilger and Diane, 2003

Euploca procumbens (Mill.) Diane & Hilger, in Hilger and Diane: Bot. Jahrb. Syst. 125 (1): 48. 2003.
Heliotropium procumbens Mill., Gard. Dict. ed. 8, Heliotropium n°10. 1768.
Euploca procumbens Type. Jamaica. *herb. Miller (coll. Houston?) s.n.* (lectotype, designated by Howard 1989, pg. 204: BM n.v. [photo GH!]).
Heliotropium americanum Mill., Gard. Dict. ed. 8, Heliotropium n°11. 1768.
Euploca procumbens Type. Mexico. Vera Cruz, 1731, *Houston s.n.* (lectotype, designated by Förther 1998, pg. 178: BM n.v.).
Heliotropium inundatum Sw., Prodr. 40. 1788.
Euploca procumbens Type (fide Förther 1998: 201). Jamaica. “insulae caribaeae”, probably a *Swartz coll.* (holotype S?).
Heliotropium decumbens Lehm., Neue Schr. Naturf. Ges. Halle 3(2): 16. 1817. Type. Venezuela. Sucre: Cumana, *Humboldt and Bonpland 57* (lectotype, designated by Förther 1998, pg. 190; B-WILLD 3239! [scan!]; isotypes MEL n.v., HAL n.v. [scan!], P! [2 sheets; scans!], P-Bonpl!) [same type collection as Heliotropium procumbens Kunth (below), but different holotype].
Heliotropium canescens Lehm., Pl. Asperif. Nucif. 38. 1818; illegitimate: not Moench 1794.
Euploca procumbens Type. Brazil. *Sieber pro Hoffmannsegg s.n.* (holotype B-WILLD 3242 n.v.; isotypes MEL 233279 n.v., HAL 71587 n.v. [scan!], P! [scan!], S n.v. [scan!]).
Heliotropium procumbens Kunth, Nov. Gen. Sp. (quarto ed.) 3: 88. 1818; nom. illeg. non Mill. 1768 + homotypic (different holo-) with Heliotropium decumbens Lehm. 1817. Type. Venezuela. Sucre: Cumana, *Humboldt and Bonpland 57* (holotype P-Bonpl!; isotypes MEL n.v., HAL n.v., P! [2 sheets], B-WILLD 3239!) [same type collection as Heliotropium decumbens Lehm. (above), but different holotype].
Heliotropium canescens Kunth, Nov. Gen. Sp. (quarto ed.) 3: 88. 1818; nom. illeg. non Moench. 1794: 415.
Euploca procumbens Type. Venezuela. near Cumana, *Humboldt 58* (holotype P!; isotype B-WILLD 3240/1!).
Heliotropium cinereum Kunth, Nov. Gen. Sp. (quarto ed.) 3: 89. 1818. Type. Venezuela. [Apure:] bank of the Apures, propre Arichuna, Mar, *Bonpland and Humboldt 1202* (holotype P-Bonpl.!; isotypes B-WILLD 3240/2!, F n.v., P! [2 sheets]). [Same type collection as Heliotropium humboldtianum below, but different holotype]
Heliotropium humboldtianum Roem. & Schult., Syst. Veg. 4: 737. 1819. Type. Venezuela. [Apure:] bank of the Apures, propre Arichuna, Mar, *Bonpland and Humboldt 1202* (lectotype, designated by Förther 1998, pg. 200: B-WILLD 3240/2!; isotypes F n.v., P! [2 sheets], P-Bonpl!). [Same type collection as Heliotropium cinereum above, but different holotype]
Heliotropium brasilianum Roth, Nov. Pl. Sp.: 103. 1821.
Euploca procumbens Type. Brazil. *Mertens s.n.* (holotype B † [photo F-17314, GH, NY, US]).
Heliotropium riparium Mart. ex Colla, Herb. Pedem. 4: 226. 1835.
Euploca procumbens Type. Brazil. Rio Belmonte, *Martius s.n.* (holotype TO n.v.; isotypes BM n.v., BR n.v., K!).
Heliotropium willdenowii G. Don, Gen. Hist. 4: 359. 1838. Type. A renaming of illegitimate Heliotropium canescens Lehm.
Heliotropium rigidulum DC., Prodr. 9: 543. 1845.
Euploca procumbens Type. Mexico. near Matamoros, July 1831, *Berlandier pl. exs. 234* (holotype G-DC n.v.; isotypes BM n.v., BP n.v., F n.v. [scan!], G-DC n.v., LE n.v., P! [2 sheets, scans!], W n.v.).
Heliotropium houstonii DC., Prodr. 9: 549. 1845; nom. illeg. renaming of Heliotropium procumbens Mill. Type. Based on Heliotropium procumbens Mill.
Schleidenia elliptica Fresen., Fl. Bras. 8(1): 42. 1857.
Euploca procumbens Type. Brazil. Minas Gerais: near Salvado, Aug 1819, *Martius s.n.* (lectotype, designated by Förther 1998, pg. 235: M n.v.; isotype M n.v.).
Schleidenia longepetiolata Fresen., Fl. Bras. 8(1): 42. 1857.
Euploca procumbens Type. Brazil. Goyaz: Rio Maranhao, *Pohl 2352* and *2384* (sic! distributed as 1593) (lectotype, designated by Förther 1998, pg. 236: W n.v. [photo F-31909, GH, US]; isotypes F n.v. [scan!; photo F-57726], K! [scan!], W n.v.).
Schleidenia inundata (Sw.) Fresen., Fl. Bras. 8(1): 43. 1857. Type. Based on Heliotropium inundatum Sw.
Schleidenia leptostachya Fresen., Fl. Bras. 8(1): 43. 1857.
Euploca procumbens Syntype: Brazil. Bahia: *Blanchet 3610* (G n.v., GH n.v. [fragments, scan!], NY!, P! [3 sheets, scans!]). Type. Brazil. Bahia: near Joazeiro, Apr 1819, *Martius s.n.* (lectotype, designated by Förther 1998, pg. 235: M [photo F-20310, NY, GH]; isotypes FI-W!, GH [fragment], M [2 sheets]).
Heliotropium ellipticum (Fresen.) Gürke, in Engler and Prantl, Nat. Pflanzenfam.. 4(3a): 97. 1893. Type. Based on Schleidenia elliptica Fresen.
Heliotropium leptostachyum (Fresen.) Gürke, in Engler and Prantl, Nat. Pflanzenfam. 4(3a): 97. 1893. Type. Based on Schleidenia leptostachya Fresen.
Heliotropium longepetiolatum (Fresen.) Gürke, in Engler and Prantl, Nat. Pflanzenfam. 4(3a): 97. 1893. Type. Based on Schleidenia longepetiolata Fresen.
Heliotropium bridgesii Rusby, Mem. Torrey Bot. Club 4: 224. 1895.
Euploca procumbens Type. Bolivia. Cochabamba, 1891, *Bang 950* (holotype NY!; isotypes B †, BM n.v., GH n.v., K n.v., LE n.v., M n.v., MO n.v. [scan!], NY!, US! [2 sheets], W n.v.).
Heliotropium riparium Chodat, Bull. Herb. Boiss., sér. 2, 2: 817. 1902; nom. illeg. non Mart. ex Colla 1835.
Euploca procumbens Type. Paraguay. “ad ripam lacus Ypacaray”, *Hassler 3893* (holotype G!; isotype P§

#### Type.

Based on *Heliotropium procumbens* Mill.

#### Description.

Annual herbs up to 50 cm long, usually cinereous; stems erect or decumbent, sericeous, trichomes appressed. Leaves: petiole slender, 0.4–2.4 cm long; lamina elliptic, obovate or broadly oblanceolate, 1–6 × 0.3–2 cm, apex acute, mucronate, base attenuate, margin entire, both surfaces sericeous. Inflorescences terminal or axillary, peduncle 1–3 cm long, slender scorpioid cymes, mostly geminate or ternate, bractless, up to 10 cm long, sericeous. Flower subsessile: calyx deeply lobed, lobes unequal, lanceolate to linear, about 2/3 as long as corolla tube; corolla white, tube 1–1.4 mm long, throat yellow, lobes spreading ovate with rounded sinus, < 0.5 mm long; stamens subsessile, anthers ovate with a small apical appendage, free; ovary about 0.3 mm diam., style lacking; stigma sessile forming with the ovary a blunt cone. Fruit: depressed-globose, 4-lobed, strigose; nutlets up to 1 mm long.

#### Distribution.

Venezuela (Amazonas, Bolívar, Delta Amacuro), Guyana, Brazil (Pará, Roraima); also West Indies and from Southern United States to Argentina.

#### Selected specimens studied.

**Guyana**. s.l., *Appun 1762* (K); 1839, *Rob. Schomburgk 1024* (K); *Schomburgk 1026* (K); Upper Takutu-Upper Essequibo, NW Kanuku Mtns, near Nappi Village, 13 Feb 1993, *Hoffman 3743* (BRG, F, L, US).

#### Note.

Förther (1998: 200) placed *Heliotropium humboltianum* Roem. & Schult. 1819 in the synonymy of *Heliotropium procumbens*. He designated as the lectotype *Humboldt and Bonpland [1202*]. *Humboldt 1202* is the type of *Heliotropium filiforme* (see above under *Euploca filiformis*).

### Names not validly published

#### Messerschmidia incana

Taxon classificationPlantaeBoraginalesHeliotropiaceae

G. Mey. 1818 nom. inval.

Messerschmidia incana G. Mey., Prim. Fl. Esseq. 92. 1818, nom. inval.

##### Type.

not designated; no original material found.

##### Note.

The only species known from the Guianas that would agree with Meyer’s description is *Euploca humilis*. Förther (1998: 238) lists *Messerschmidia incana* as a synonym of *Heliotropium ternatum* [=*Euploca humilis*] with a question mark, but Meyer placed a synonym in the protolog of his new species, *Tournefortia sessilifolia* Poir. (1804: 360), a species described from Argentina. Because *sessilifolia* was available in *Messerschmidia*, it should have been used as the epithet. Therefore the name by Meyer is not validly published.

## Supplementary Material

XML Treatment for Euploca

XML Treatment for Euploca foliata

XML Treatment for Euploca humilis

XML Treatment for Euploca filiformis

XML Treatment for Euploca humilis

XML Treatment for Euploca humistrata

XML Treatment for Euploca lagoensis

XML Treatment for Euploca polyphylla

XML Treatment for Euploca procumbens

XML Treatment for Messerschmidia incana

## List of taxa

***Euploca*** Nutt. 1836

***Euploca convolvulacea*** Nutt. (type of ***Euploca***)

***Euploca filiformis*** (Lehm.) J.I.M.Melo & Semir 2009

***Euploca foliata*** (R. Br.) Feuillet **comb. nov.**

***Euploca humilis*** (L.) Feuillet, **comb. nov.**

***Euploca humistrata*** (Cham.) J.I.M.Melo & Semir 2009

***Euploca hypogaea*** (Urb. & Ekman) Diane & Hilger

***Euploca lagoensis*** (Warm.) Diane & Hilger 2003

***Euploca paradoxa*** (Mart.) J.I.M.Melo & Semir

***Euploca polyphylla*** (Lehm.) J.I.M.Melo & Semir 2009

***Euploca procumbens*** (Mill.) Diane & Hilger 2003

*Euploca ternata* (Vahl) J.I.M.Melo & Semir 2009 = ***Euploca humilis***

*Heliophytum passerinoides* Klotzsch 1849 = ***Euploca humilis***

Heliotropium subgen. Orthostachys (R.Br.) Rchb. 1841 = ***Euploca***

Heliotropium sect. Orthostachys R.Br. 1810 = ***Euploca***

Heliotropium sect. Orthostachys subsect. Axillaria I.M.Johnst. 1928 = ***Euploca***

*Heliotropium americanum* Mill. 1768 = ***Euploca procumbens***

*Heliotropium bahiense* DC. 1845 = ***Euploca polyphylla***

*Heliotropium brasilianum* Roth 1821 = ***Euploca procumbens***

*Heliotropium bridgesii* Rusby 1895 = ***Euploca procumbens***

*Heliotropium canescens* Kunth 1818 = ***Euploca procumbens***

*Heliotropium canescens* Lehm. 1818 = ***Euploca procumbens***

*Heliotropium cinereum* Kunth 1818 = ***Euploca procumbens***

*Heliotropium decumbens* Lehm. 1817 = ***Euploca procumbens***

*Heliotropium ellipticum* (Fresen.) Gürke 1893 = ***Euploca procumbens***

*Heliotropium filiforme* Kunth 1818 = ***Euploca filiformis***

*Heliotropium filiforme* Lehm. 1817 = ***Euploca filiformis***

*Heliotropium foliatum* R.Br. 1810 (type of Heliotropium sect. Orthostachys (R.Br.) G.Don) = ***Euploca foliata***

*Heliotropium foliosum* Roem. & Schult. 1819 = ***Euploca polyphylla***

Heliotropium fruticosum var. ternatum (Vahl) DC. 1845 = ***Euploca humilis***

*Heliotropium fumana* (Fresen.) Gürke 1893 = ***Euploca humilis***

*Heliotropium greenmanii* Wiggins 1950. = ***Euploca humilis***

*Heliotropium helophilum* Mart. 1838 = ***Euploca filiformis***

*Heliotropium hirtum* Lehm. 1817 = ***Euploca humilis***

*Heliotropium hispidum* Kunth 1818 = **Euploca humilis**

*Heliotropium horizontale* Small, Bull. 1905 = ***Euploca polyphylla***

*Heliotropium houstonii* DC. 1845 = ***Euploca procumbens***

*Heliotropium humboldtianum* Roem. & Schult. 1819 = ***Euploca procumbens***

*Heliotropium humboltianum* Roem. & Schult. 1819 = ***Euploca procumbens***

*Heliotropium humistratum* Cham. 1829 = ***Euploca humistrata***

*Heliotropium inundatum* Sw. 1788 = ***Euploca procumbens***

*Heliotropium laevenworthii* (A. Gray) Small 1903 = ***Euploca polyphylla***

*Heliotropium lagoense* (Warm.) Gürke 1893 = ***Euploca lagoensis***

*Heliotropium leptostachyum* (Fresen.) Gürke 1893 = ***Euploca procumbens***

*Heliotropium linifolium* (A. St.-Hil.) Gürke 1893 = ***Euploca polyphylla***

*Heliotropium littorale* Mart. ex Colla 1835 = ***Euploca filiformis*** (Lehm.) J.I.M.Melo & Semir

*Heliotropium longepetiolatum* (Fresen.) Gürke 1893 = ***Euploca procumbens***

*Heliotropium mexicanum* Greenm. 1898 = ***Euploca humilis***

*Heliotropium oaxacanum* DC. 1845 = ***Euploca humilis***

*Heliotropium paradoxum* (Mart.) Gürke 1893 = ***Euploca paradoxa***

*Heliotropium polyphyllum* Lehm. 1817 = ***Euploca polyphylla***

Heliotropium polyphyllum var. blanchetii DC. 1845 = ***Euploca polyphylla***

Heliotropium polyphyllum var. horizontale (Small) R.W. Long 1970 = ***Euploca polyphylla***

Heliotropium polyphyllum var. laevenworthii A. Gray 1878 = ***Euploca polyphylla***

*Heliotropium procumbens* Kunth 1818 = ***Euploca procumbens***

*Heliotropium procumbens* Mill. 1768 = ***Euploca procumbens***

*Heliotropium pubescens* (Fresen.) Gürke 1893 = ***Euploca polyphylla***

*Heliotropium pusillum* Colla 1835 = ***Euploca filiformis***

*Heliotropium rigidulum* DC. 1845. = ***Euploca procumbens***

*Heliotropium riparium* Chodat 1902 = ***Euploca procumbens***

*Heliotropium riparium* Mart. ex Colla 1835 = ***Euploca procumbens***

Heliotropium sect. Euploca (Nutt.) A. Gray 1874 = ***Euploca***

*Heliotropium stenostachyum* (A. St.-Hil.) Gürke 1893 = ***Euploca filiformis***

*Heliotropium strictissimum* sensu N.E. Br. 1901 = ***Euploca humilis***

*Heliotropium tenue* Roem. & Schult. 1819 = ***Euploca filiformis***

*Heliotropium ternatum* Vahl 1794 = ***Euploca humilis***

Heliotropium ternatum var. fumana (Fresen.) I.M. Johnst. 1935 = ***Euploca humilis***

*Heliotropium trinitense* Urb. 1912 = ***Euploca lagoensis***

*Heliotropium willdenowii* G. Don 1838 = ***Euploca procumbens***

*Hilgeria* Förther 1989 = ***Euploca***

*Hilgeria hypogaea* (Urb. & Ekman) Förther 1989 (type of *Hilgeria* Förther) = ***Euploca hypogaea***

*Messerschmidia incana* G. Mey. 1818 = ***Euploca humilis***

*Orthostachys* (R. Br.) Spach 1840 = ***Euploca***

*Pioctonon antillanum* Raf. 1838 = ***Euploca humilis***

*Pioctonon ternatum* (Vahl) Raf. 1838 = ***Euploca humilis***

*Preslaea* Mart. 1827 = ***Euploca***

*Preslaea linifolia* A. St.-Hil. 1833 = ***Euploca polyphylla***

*Preslaea paradoxa* Mart. 1827 (type of *Preslaea* Mart.) = ***Euploca paradoxa***

*Preslaea stenostachya* A. St.-Hil. 1833 = ***Euploca filiformis***

*Schleidenia* Endl. 1839 = ***Euploca***

*Schleidenia bahiensis* (DC.) Fresen. 1857 = ***Euploca polyphylla***

*Schleidenia elliptica* Fresen. 1857 = ***Euploca procumbens***

*Schleidenia filiformis* (Lehm.) Fresen. 1857 = ***Euploca filiformis***

*Schleidenia fumana* Fresen. 1857 = ***Euploca humilis***

*Schleidenia hispida* (Kunth) Fresen. 1857 = ***Euploca humilis***

*Schleidenia humistrata* (Cham.) Fresen. 1857 = ***Euploca humistrata***

*Schleidenia inundata* (Sw.) Fresen. 1857 = ***Euploca procumbens***

*Schleidenia lagoensis* Warm. 1868 = ***Euploca lagoensis***

*Schleidenia leptostachya* Fresen. 1857 = ***Euploca procumbens***

*Schleidenia linifolia* (A. St.-Hil.) DC. 1845 = ***Euploca polyphylla***

*Schleidenia longepetiolata* Fresen. 1857 = ***Euploca procumbens***

*Schleidenia polyphylla* (Lehm.) Fresen. 1857 = ***Euploca polyphylla***

Schleidenia polyphylla var. blanchetii (DC.) Fresen. 1857 = ***Euploca polyphylla***

*Schleidenia pubescens* Fresen. 1857 = ***Euploca polyphylla***

*Schleidenia stenostachya* (A. St.-Hil.) DC. 1845 = ***Euploca filiformis***

*Schleidenia subracemosa* Warm. 1868 = ***Euploca lagoensis***

*Tournefortia humilis* L. 1753 = ***Euploca humilis***

*Tournefortia incana* (G. Mey.) G. Don 1838 = ***Euploca humilis***

*Tournefortia meyeri* DC. 1845 = ***Euploca humilis***
